# Invited Article: Emerging soft bioelectronics for cardiac health diagnosis and treatment

**DOI:** 10.1063/1.5060270

**Published:** 2019-03-01

**Authors:** Faheem Ershad, Kyoseung Sim, Anish Thukral, Yu Shrike Zhang, Cunjiang Yu

**Affiliations:** 1 Department of Biomedical Engineering, University of Houston, Houston, Texas 77204, USA; 2 Department of Mechanical Engineering, University of Houston, Houston, Texas 77204, USA; 3 Materials Science and Engineering Program, University of Houston, Houston, Texas 77204, USA; 4 Division of Engineering in Medicine, Department of Medicine, Brigham and Women’s Hospital, Harvard Medical School, Cambridge, Massachusetts 02139, USA; 5 Department of Electrical and Computer Engineering, University of Houston, Houston, Texas 77204, USA

## Abstract

Cardiovascular diseases are among the leading causes of death worldwide. Conventional technologies for diagnosing and treating lack the compliance and comfort necessary for those living with life-threatening conditions. Soft electronics presents a promising outlet for conformal, flexible, and stretchable devices that can overcome the mechanical mismatch that is often associated with conventional technologies. Here, we review the various methods in which electronics have been made flexible and stretchable, to better interface with the human body, both externally with the skin and internally with the outer surface of the heart. Then, we review soft, wearable, noninvasive heart monitors designed to be attached to the chest or other parts of the body for mechano-acoustic and electrophysiological sensing. A common method of treatment for various abnormal heart rhythms involves catheter ablation procedures and we review the current soft bioelectronics that can be placed on the balloon or head of the catheter. Cardiac mapping is integral to determine the state of the heart; we discuss the various parameters for sensing aside from electrophysiological sensing, such as temperature, pH, strain, and tactile sensing. Finally, we review the soft devices that harvest energy from the natural and spontaneous beating of the heart by converting its mechanical motion into electrical energy to power implants.

The heart is the body’s mechanical pump, constantly pushing oxygenated and deoxygenated blood throughout the body as it endlessly contracts and relaxes the atria and ventricles. As one of the most vital organs of the body, the heart must be continuously monitored for those afflicted by heart diseases. Nearly 41 × 10^6^ individuals globally have experienced heart failure.^1,2^ Greater than 5 × 10^6^ individuals are affected (in the developed world alone) by atrial fibrillation (AF) specifically, which has been notorious for stroke in patients with cardiac diseases.^3,4^ The number of patients with complex arrhythmias is expected to rapidly increase in the near future.^5,6^ To better understand the origins of heart diseases and clearly corroborate the need for soft electronics in cardiac sensing, the mechanism of blood flow and the electrical conduction system of the heart are detailed.

The cardiac cycle consists of two phases: systole, the contraction of the heart to pump blood, and diastole, the relaxation of the heart after contraction. General blood flow in the heart is as follows. Diastole involves the movement of blood lacking in oxygen flowing in from the vena cava to the right atrium, in which the chordae tendineae open the tricuspid valve.^7^ The right atrium contracts, causing the deoxygenated blood to flow into the right ventricle (RV), which subsequently contracts (now in systole) and pushes the blood through the pulmonary artery. This blood is transported to the lungs. Blood returning from the lungs (oxygenated) through the pulmonary vein enters the left atrium, which contracts and pushes blood through the bicuspid (or mitral) valve, into the left ventricle.^7^ This ventricle contracts (systole), directing blood through the aortic valve and then to the aorta, which is responsible for distributing nutrient-rich blood to the rest of the body. The valves that operate in the heart open/close in response to gradients in pressure.^7^ Ascertaining proper blood flow is critical in diseased patients and numerous fatal conditions can arise, aside from myocardial infarction. Systolic heart failure can occur if the pressure in the left ventricle is not enough to close the mitral valve.^8^ If the mitral valve does not close properly, then regurgitation, or backward flow of blood, from left ventricular contraction can occur.^9^ Aortic stenosis can also lead to improper blood flow as the heart must pump more to push blood through a narrowed aortic valve.^10^ Ischemia, or inadequate supply of blood to organs or parts of the body, can occur if blood flow is impeded.^11^ Thrombosis and/or embolization (blood clotting) can significantly hinder blood flow as well. During beating, the heart expands to 145% of its contraction volume and the inner chambers have been shown to place 20%-30% strain on interfaced electronics.^12,13^

The mechanism of contraction and relaxation occurs in conjunction with a special conduction system that maintains the persistent beating of the heart [Fig. 1(a)]. Cardiomyocytes are the excitable cells which compose the cardiac muscle (atria and ventricles) while cardiac pacemaker cells initiate the electrical impulse. Between all these cells are intercalated discs (junctions) that bridge the cells and allow for the propagation of electrical impulses from one cell to another. These discs allow for the passage of various ions that are responsible for the depolarization and repolarization events of the heart, which are recordable and visualized in the electrocardiogram [Fig. 1(b)]. The sinoatrial (SA) node, located in the right atrium, contains pacemaker cells; the heart rate directly depends on the rate of the action potential produced by these cells. The pacemaker cells spontaneously depolarize, and this depolarization spreads through the atria. Depolarization at the SA node and in the right atrium corresponds to the P wave of the typical electrocardiography (ECG) waveform and occurs due to the flow of sodium ions into the cells. After depolarization, sodium continues to enter the cells resulting in an overshoot, which leads to the sodium channels’ closing and calcium channels’ opening, once the membrane potential of the cell reaches a certain threshold. The impulse from the SA node propagates to the atrioventricular (AV) node. From the AV node, the impulse travels through the bundle of His and eventually reaches the Purkinje fibers, which contact the ventricular cardiomyocytes. Following the depolarization of the atrium is the depolarization of the ventricles and repolarization of the atria; this can be observed with the characteristic QRS peak found in the ECG waveform. Finally, the T wave in the ECG represents the repolarization of the ventricles. From the ECG, various diseases can be diagnosed, though a majority of these diseases form a class of diseases known as arrythmias, or irregular beating of the heart.^14^ Some examples are P wave asystole (or no ventricular activity/blood flow, only P wave in ECG), tachycardias (abnormally high heart rate, only QRS is shown or lengthened), ventricular fibrillation (inconsistent and fast beating, no identifiable wave can be observed), atrial fibrillation (inconsistent heartbeats, no pattern between QRS complexes and no P wave), or other arrhythmias. The information that can be provided from mechano-acoustic and electrophysiological signals is discussed below.

**FIG. 1.**
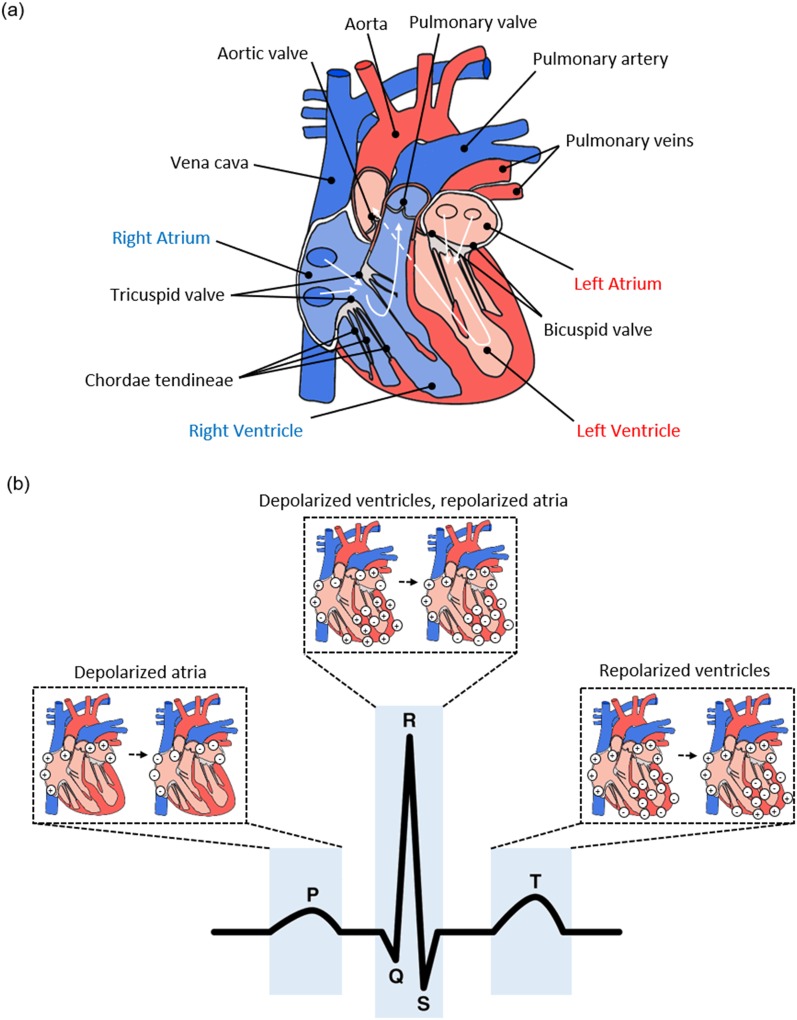
Mechanism of blood flow (a) with arrows indicating the direction of blood flow in the heart. Light blue regions indicate areas through which deoxygenated blood flows, while pink regions indicate where oxygenated blood flows.^7^ The origin of the characteristic electrocardiography waveform (b) with the major depolarizations from which the sub-waves (P, QRS, T) arise.^7^

Mechano-acoustic signals can disclose information about heart sounds, which are critical to determine the proper heart function. Heart sounds indicate the closure of heart valves, although they are ∼30 mm beneath the surface of the chest. Still, heart sounds can be used to verify correct blood flow between the chambers of the heart, even from the skin.^15^ Methods of measuring these types of signals include ballistocardiography (BCG, a measure of recoil forces from the body as the heart pumps blood through blood vessels), phonocardiography (PCG, a measure of sounds the heart makes), and seismocardiography (SCG, a measure of chest vibrations due to heartbeat).^16^ Photoplethysmography (PPG) is used to measure the changes in blood volume in peripheral blood vessels, and it provides information about the timing of cardiac cycles. PPG has been used in place of ECG in some cases although it has limited signal accuracy.^17–20^ In addition to these measurement techniques, ventricular assist devices have been developed to act as support systems for those who have experienced heart failure; they are mechanical pumps that help the heart to pump blood from the ventricles to the rest of the body. Mechano-acoustic signals can supplement the information provided by ECG and can sometimes even present information that cannot be directly found in ECG, specifically in the case of heart sounds.^15^ Continuing to the electrophysiological side of diagnosis, ECG is a critical tool for both noninvasively and invasively diagnosing many disorders associated with the heart. By understanding the mechanism behind the production of the ECG signal, it is possible to determine abnormal heart conditions by observing the signals found in the ECG. Holter monitoring devices with multiple leads are the current clinical standard for recording ECG.^21–25^ One common method of treatment for abnormal heart rhythms is radiofrequency ablation. Cells that are thought to prevent the appropriate propagation of the impulse through the conduction pathway in the heart are destroyed by heating or freezing using catheters. In addition to characterizing the blood flow and electrical activity of the heart, temperature, strain, and pH are other vital parameters that should be monitored.

Accelerometers have been used in bulky/rigid formats to detect mechano-acoustic signals.^16^ However, these have usually been strapped to the user. In addition to discomfort from the mechanical coupling of the device with skin, the conventional technology could have reduced sensitivity to more subtle movements that have physiological consequences, exist in large form factors which prevent optimal placement of the device, and only perform single-mode sensing. Typically, rigid ventricular assist devices (VADs) are used to work in place of one or both ventricles of the heart for patients who are waiting for heart transplants. However, since they create artificial surfaces in the body, they put patients at a greater risk of stroke and thromboembolic events; blood-thinning medications need to be offered to the patients requiring VADs.^26–29^ External devices have been shown to work against the native cardiac mechanics and but they cannot always fully synchronize with cardiac contraction.^30^ These sensors, as well as ECG monitors, must both be considered.

Current ECG monitors lack full compliance with the human body.^31–34^ Rigid, flat substrates were commonly used for recording ECG in isolated cell culture studies and mapping impulse propagation.^35–39^ Although some recent technologies exploit flexible substrates, the measurement interface is not always optimal.^12,40–45^ Skin-mounted, soft, and imperceptible devices have been shown to be more compliant while also providing greater details about cardiac health.^46^ A survey from a recent study shows that patients overwhelmingly prefer a skin-mounted and flexible sensor over the conventionally used Holter monitor for comfortable daily usage.^47^ In addition, there are needs for other types of sensors (such as pH sensors and tactile sensors) to be implemented in flexible and stretchable formats.^48,49^

In order for bioelectronics to intimately interface with the beating heart or skin, they need to be designed and constructed into flexible and stretchable formats. In other words, they must comprise flexibility and/or stretchability. Various strategies exist to induce these two characteristics and the underlying physics has been studied extensively.^50–52^ The requirement for mechanical flexibility usually can be met by making the materials and devices thin. For example, consider the case of plastics, such as polyimide (PI), a commonly utilized substrate for flexible electronics, the bending stiffness is directly proportional to the cubic power of the thickness of the material.^53,54^ Thus, reducing the thickness of the substrate by a factor of 10 will lead to a bending stiffness reduced by a factor of 1000. If a device is sufficiently bendable or flexible and minimizes the conformal energy, then it can achieve conformal contact with the surface of the skin or heart.

For the case of electronics that are compliant with the human skin, conformal contact can be achieved if the device is less than 3.8-*µ*m thick; this is due to sufficiently low contact pressure on skin with average roughness.^54^ But again, this also depends on the roughness of the skin, bending energy of the substrate, and interfacial adhesion energy.^54–56^ The rougher the skin is, the thinner the device will need to be to account for the higher interfacial pressure. Some examples of conformal and flexible electronics are shown in Figs. 2(a) and 2(b).

**FIG. 2.**
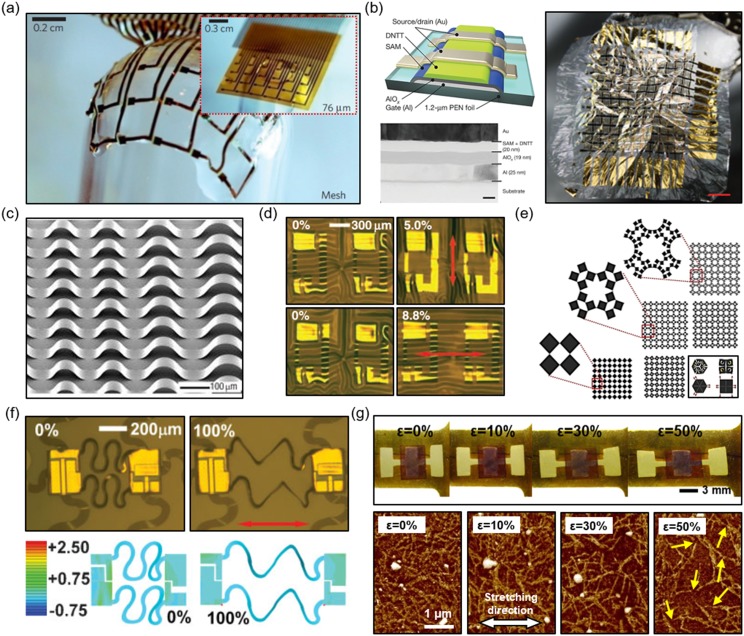
Examples of soft electronics as shown in (a) thin film mesh (thickness: 2.5 *µ*m) reduces adhesion energy and allows for the device to wrap over a glass beam, the inset shows the device in an unwrapped state, but it is too thick to conform to the surface;^55^ (b) ultra-flexible thin plastic electronics: the left panel shows the structure of the thin film transistor (scale bar, 20 nm), and the right panel shows the device crumpled like paper (scale bar, 1 cm);^56^ (c) controlled wrinkling of stretchable Si ribbons on 50% pre-strained PDMS;^58^ (d) stretchable Si-based complementary metal-oxide semiconductor (CMOS) electronics on PDMS, images show tensile strains on the device in vertical (top) and horizontal (bottom) directions;^63^ (e) kirigami structures showing in-plane stretchability, % stretch from bottom to top row: 43%, 62%, and 79%, and the inset (bottom right) shows how kirigami structures rotate and stretch;^70^ (f) upper frames show the CMOS inverter and lower frames show finite element modeling of stretched interconnects;^71^ and (g) intrinsically stretchable semiconductor [poly(3-hexylthiophene-2,5-diyl), P3HT, in PDMS] based sensor at different strains in top frames and the corresponding AFM phase mode image of P3HT fibers in bottom frames.^84^

To induce stretchability, a more complex characteristic to achieve than flexibility, multiple strategies have been implemented. Most of the strategies for stretchability can be simplified into two approaches. For conventionally non-stretchable materials, forms of structural engineering are required, while the other approach involves utilizing intrinsically stretchable materials. In making non-stretchable materials stretchable, one strategy involves creating buckled/wavy structures and taking advantage of out-of-plane motion to account for in-plane strain.^57,58^ By bonding thin layers of materials to an elastomeric substrate, such as polydimethylsiloxane (PDMS), that undergoes pre-strain and then is released, a wavy structure with distinct amplitudes and wavelengths can be created.^59–61^ Figures 2(c) and 2(d) show the success of this strategy to develop stretchable electronics from rigid Si.

Altering the material properties and pre-strain can tune the amplitude and wavelength of the wavy structure. With greater pre-strain, larger amplitude waves with shorter wavelengths can be formed. Like in an accordion, the wavy structures experience changes in their wavelength and amplitude to account for stretching.^62^ Instead of exploiting out-of-plane wavy structures, in-plane wavy or deformable structures have also been employed to provide mechanical stretchability.^63–68^ For instance, stretchable serpentine interconnects as bridges can be formed to connect strain-sensitive electronics as islands that are bonded to the substrate.^69^ This is an effective method to delocalize strain away from the passive/active sensing components of a given device. Other strategies for in-plane wavy and deformable structures have been realized as well [Figs. 2(e) and 2(f)].^70,71^ These include coiled springs with rotatable islands, leaf-arm springs, and serpentine-shaped interconnects.^72–81^ Using fractal architecture is another strategy for attaining in-plane stretchability.^82^ Advanced methods can be used to allow for twisting, rotating, bending, and buckling both in-plane and out-of-plane.^69^ Kirigami, which involves strategically creating arrays of cuts on a material, is a concept that has been implemented to enable reduced stress in buckling/folding applications, and also for providing stretchability both in-plane and out-of-plane.^51,69^ For interfacing with the heart, in-plane structures may be more beneficial [Fig. 2(e)]. Implementing intrinsically stretchable materials on elastomeric substrates is another common approach for achieving stretchability as structural engineering can be complicated and costly.^83^ Although the development for fully intrinsically stretchable devices that combine intrinsically stretchable conductors, dielectrics, and semiconductor materials is in progress, individual components have been studied [Fig. 2(g)].^84,85^ Intrinsically stretchable interconnects, electrodes, and piezoresistive sensors are the most investigated components so far.^86–91^

An emerging class of bioelectronics, namely, epidermal electronics, which can be skin-mountable, compliant, and even mechanically imperceptible to the wearers, overcomes the outstanding challenge of mechanical mismatches between electronics and the skin.^92^ Primarily, these sensors are used for monitoring the electrical potentials generated during the heartbeat at the surface of the skin. In some applications, these sensors are capable of acting as stethoscopes, a ubiquitous tool for analyzing heart sounds found in all physician’s offices today. The studies here present various devices and their applications for noninvasive heart monitoring.

Liu *et al.* fabricated mechano-acoustic and electrophysiological sensors on a single device platform using stretchable electronics that conform to skin [Fig. 3(a)].^15^ The devices were 2 mm thick, have moduli of ∼31 kPa, and bending stiffnesses of ∼1 *µ*Nm. The sensing platform had a core/shell structure in which the core combined serpentine copper interconnects, an accelerometer, hardware filters (resistors and capacitors), and an amplifier at the neutral mechanical plane (NMP) between two layers of PI encapsulation, all of which were embedded in a soft elastomer (Silbione). Eco-flex acted as the shell. This type of structure both isolated the main components from stress at the interface of the skin and restricted the movement of interconnects. Au electrodes and an anisotropic conductive film (ACF) connector existed at openings of the core/shell devices. An additional layer of Silbione underneath the devices served as the adhesive layer. After confirming that their device was biocompatible through a cell viability test, they accounted for mechanical impedance by minimizing the mass of the device, which resulted in lower mechanical loading at the skin. They mounted the device onto the sternum of a healthy human patient and a diseased patient, for simultaneous SCG and ECG measurements [Figs. 3(b) and 3(c)]. For the healthy patient, it was observed that the mechano-acoustic signals were similar in quality to those of the conventional stethoscope. For diseased patients who were diagnosed with cardiac stenosis/regurgitation, the device captured abnormal heart sounds at the pulmonary and tricuspid areas. Finally, the mechano-acoustic device was laminated onto a left ventricular assist device (LVAD). LVADs are intended to be temporary solutions for those waiting for heart transplants. In a simulated experiment, a blood clot was entered into the air valve of the LVAD, and the mechano-acoustic device registered additional abnormal frequencies on top of the frequencies associated with regular LVAD operation. In addition to local heart monitoring, remote monitoring can be incredibly beneficial to patients.

**FIG. 3.**
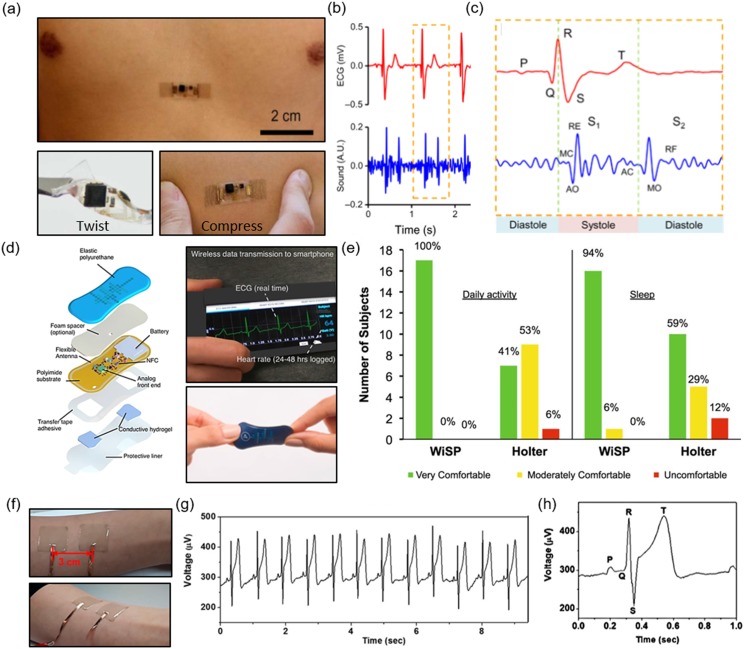
Mechano-acoustic sensor on (a) the sternum, lower frames show twisting and compression of the device;^15^ (b) ECG and heart sounds recorded from the sensor;^15^ (c) zoomed in from (b) showing sub-waves of ECG; MC, mitral valve closed; AO, aortic valve opened; RE, rapid ejection from ventricle; AC, aortic valve closed; MO, mitral valve opened; RF, rapid filling of ventricles; time between S_1_ and S_2_ is the systolic time of left ventricle;^15^ (d) schematic of the WiSP device, right frames shows wireless data transmission (top) and twisting (bottom);^47^ (e) survey data from a hospital comparing comfortability of the WiSP and Holter monitors;^47^ (f) Ag embedded in adhesive PDMS electrodes on skin;^93^ (g) recordings from electrodes in (f);^93^ and (h) zoomed in recording of (g).^93^

Lee *et al.* created a bandage-like device (referred to as WiSP from here on) that is flexible, wearable, and used for remote monitoring of the heart.^47^ The WiSP epidermal sensor logged heart rate, ECG, and harvested energy for power through near-field communication, while it also had its own custom battery. In addition, the sensor wirelessly streamed data to smartphones with near-field communication (NFC) capabilities [Fig. 3(d)]. Polyurethane (PU) encapsulated a flexible PI substrate which held the flexible antenna, battery, NFC chip, and circuitry. At the bottom of the device, a tape adhesive had exposed areas for Au contact pads coated with conductive hydrogel. The electrodes were spaced appropriately so that the entirety of the typical ECG waveform could be captured. The thickness of the PI was optimized to 0.2 mm from 2 mm to allow for conformal contact with skin. When a smartphone was brought within 3 cm of the WiSP, the device was activated. The smartphone was used to control the functionality of the WiSP and securely streamed data from the patient to the caregiver. For healthy subjects, the WiSP ECG waveforms were comparable in quality to those of a commercial device from all standard 3-lead ECG measurement positions on the body (lead I, II, and III). For remote monitoring of healthy subjects, they let the patients resume daily life at home with the WiSP attached to their skin. They also attached a commercial, wearable strap used for heart monitoring. The comparison of the data showed high correlation between the WiSP and commercial monitor, suggesting that the WiSP performed well during regular usage. The WiSP was then implemented in a remote monitoring study of subjects with atrial fibrillation, in which both the WiSP and Holter monitors recorded heart rate data for comparison. Atrial fibrillation episodes were evident and accurately detected from both devices. A survey taken at a hospital showed that the WiSP is more comfortable in both daily use and sleep in comparison to the Holter monitor [Fig. 3(e)]. In addition to flexible and stretchable devices, optically transparent devices could be useful for noninvasive monitoring, both functionally and aesthetically.

Park and co-workers developed stretchable electrodes with high adhesion and optical transmittance for strain sensing and ECG recording from the skin [Fig. 3(f)].^93^ The electrodes were fabricated by embedding Ag nanowires (AgNWs) in adhesive PDMS (a-PDMS) by first spin coating AgNWs on glass, then coating the AgNW with the a-PDMS, and finally releasing the film from the glass. They tuned the adhesiveness, Young’s modulus, and failure strain of PDMS by varying the weight percent of a nonionic surfactant, Triton X, when mixing with the PDMS elastomer base. Varying the curing temperature and the addition of 4 wt. % Triton (referred to as a4-PDMS) enhanced the mechanical properties of the PDMS. By differing the curing temperature, they were able to determine that a4-PDMS cured at 40 °C demonstrated the best Young’s modulus (40 kPa) and over 400% strain at failure. This is exceptional for applications on human skin, which has a modulus between 500 kPa and 2 MPa, because a lower Young’s modulus increases the interfacial contact area between the electrode and skin, leading to greater conformal attachment and more comfortable user experience. From their peeling test, the adhesion force of a4-PDMS was found to be 6-fold greater than that of unmodified PDMS. This was a result of better spreading of polymer chains and improved wetting, leading to greater surface contact. In the case of optical transparency, a4-PDMS showed greater than 80% transmittance, although it was less transparent than non-modified PDMS. Considering all the other benefits of the a4-PDMS, its optical transparency still permits it to be potentially used in optogenetic studies involving pacing experiments should the device be capable of adhering to the exterior surface of the heart.^94^ The stability of this adhesive PDMS was examined and after 2 months of storage, Young’s modulus was about 51 kPa and the failure strain was ∼400%. After embedding the AgNW (now referred to as a4-PDMS_NW), the stretchable conductor was found to have a sheet resistance of 35 Ω/sq with a transmittance of 75%. The relative resistance change was found to decrease after 2000 cycles of stretching at 15% strain; the AgNWs changed their alignment to that of the stretching direction. To examine the performance of the a4-PDMS_AgNW conductor, they compared its ECG recording capability against commercial gel electrodes. Using a three-electrode setup on the chest and ribcage, they recorded the ECG. The a4-PDMS-AgNW showed negligible noise, even less than that of the commercial electrodes. The ECG signal clearly contained the expected P, QRS, and T waves [Figs. 3(g) and 3(h)]. The clarity of the signal can be associated with the low impedance and conformal attachment of the electrodes to the skin.

Balloon catheters are powerful minimally invasive tools that can house multiple functionalities. The shaft of the catheter can be inserted through small incisions in the body. The configuration of the balloon can be controlled by the material choice. Catheters can be inserted into multiple areas of the heart, but generally, those areas are in the epicardium (inner layer of the pericardium, which is the outer layer covering the heart) or the endocardium (inner walls of the chambers in the heart). In angioplasty, these catheters can inflate coronary arteries to remove blockages, and in septostomy, the balloons provide greater force in widening the area between the atria to allow for more efficient pumping in the right side of the heart.^95–98^ Soft catheter electronics have been fabricated by releasing ultrathin metal and/or inorganic materials from their substrate onto the balloons in various ways, the main method being transfer printing.^44,99,100^ PI or epoxies bolster adhesion, allowing for many inflating/deflating cycles without loss of performance from the sensors. Special layouts of the sensing materials and/or interconnects need to be implemented to account for the 20%-30% strain created by contraction/relaxation of the heart. Catheters can also be used for radiofrequency (RF) ablation therapy, which is a technique that involves the electrical stimulation, cryoablation, or laser ablation of individual, problematic cells that cause arrhythmias like AF.^101–105^ Once these cells are destroyed, the normal conduction pathways can be restored, leading to a stabilization of the heart rate, which can be observed in the ECG. During these procedures, temperatures greater than ∼40 °C can cause tissue damage.^106^ The purpose of the soft electronics on the balloon is to conformally contact the surface and chambers of the heart, which are curvilinear. For the devices on the balloon, placement of the sensors and actuators at the NMP limits the concentration of strain on the sensors. Current balloon catheters cannot yield information about mechanical contact, local temperature, and the electrical state of the tissue.^44,99,100^ The electrodes used for conventional catheters also lack the spatial resolution needed to capture the rapid waves that compose the ECG, with electrode densities around 0.1 electrodes/cm^2^. In addition to improving blood flow, catheters can provide quantitative physiological information from the interfaced tissue, should they be equipped with the appropriate sensors; these sensors are discussed here.

Kim *et al.* deployed flexible and stretchable electronics on a multifunctional balloon catheter that surmount the limitations of conventional catheters which do not provide information regarding blood flow, contact pressure, lesion area, and temperature [Fig. 4(a)].^44^ The strain resistance of the devices can be attributed to the fact that they were placed at the nodes of the mesh, which reduced the mechanical coupling to strain as the balloon expanded and contracted and to the serpentine layouts and interconnections. This catheter consisted of micro-tactile sensors, temperature sensors, RF ablation electrodes, blood flow sensors, recording electrodes, and active semiconductor devices. The micro-tactile sensors were used to quantify the mechanical forces on the heart tissue and were in non-coplanar serpentine layouts with the sensors placed at nodes which experience minimal strain (<1%), even as the substrate underwent large deformations. They were sensitive enough to capture the normal forces without destroying the soft tissue. The sensors were located on thick layers of SU8 (epoxy) and had an electrically conductive silicon rubber (pressure-sensitive rubber, PSR) which overlaid PDMS. PI encapsulated this structure. Lateral tensile strain was shown to modulate the resistance of PSR; this was the working mechanism of the micro-tactile sensor. The temperature sensor, similar in design to the micro-tactile sensor, was made of Pt and had a resistance change of 1.91 Ω/°C; the resistance of the interconnects contributed negligibly to the measurement. Temperature sensing is critical as heat is released from current flowing between the active and ground electrodes during ablation. With this sensor, it is possible to detect the lesion size in the radial and thickness directions. Such information can greatly inform a surgeon about the progress of the surgery. The blood flow sensor detected changes in blood flow as resistance changes. With greater flow rate, the resistance generally increased. The RF ablation electrodes, electrophysiological recording electrodes, and LEDs were all also integrated into the same platform. In their *in vivo* experiment, the device was inserted into the epicardium of rat and rabbit hearts. ECG recordings showed elevated S-T intervals at the RV and some regions of the left ventricle (LV). In evaluating the S-T interval, inflation-induced injuries from the balloon were deduced. This could be imperative to understanding the contact pressures of inflating the balloon in the endocardium as well. The micro-tactile sensors also provided a method to monitor balloon inflation. *In vivo* ablation was successfully performed as they were able to control the size of the lesion using the RF ablation electrodes and temperature sensors. Finally, they modeled a disease state and demonstrated that the electrodes functioned from the onset of the injury, leading to obvious indicators of disease in the ECG. Follow-up recordings at other sites after this induced injury also showed abnormalities. The sensors presented in this highly integrated and soft platform can also be used on surgical gloves to probe more sensitive areas during cardiac surgery, such as the posterior myocardium. In another study, Klinker *et al.* developed a similar device with a blood flow sensor on a balloon catheter that also deployed stretchable electrodes for recording and ablation [Fig. 4(b)].^107^ A balloon catheter at different inflation states is shown in Fig. 4(c). Balloons catheters with higher-density electrodes could provide information that current mapping electrodes for catheters do not have the resolution to do.

**FIG. 4.**
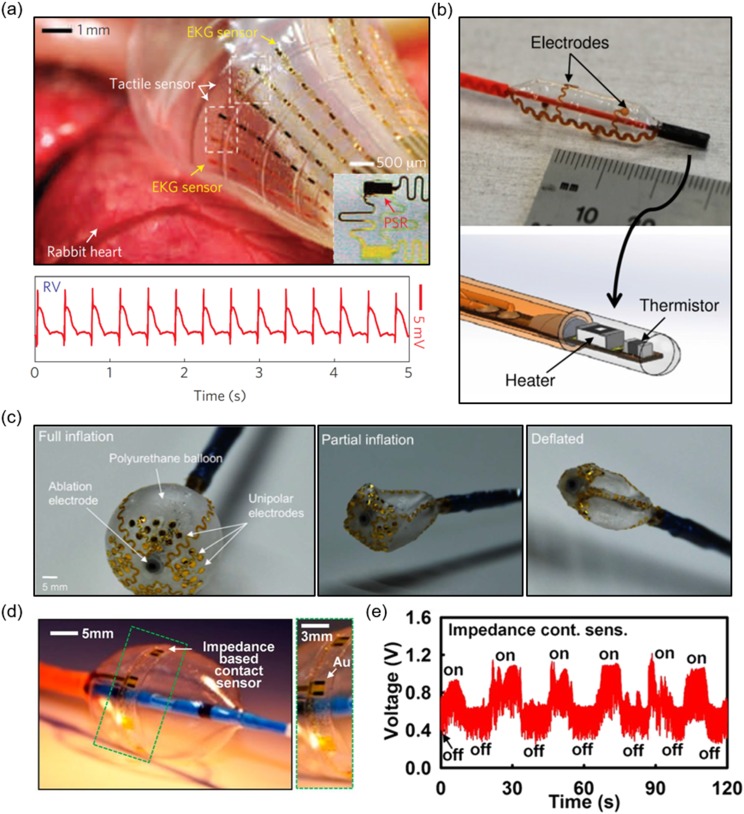
Balloon catheter with (a) stretchable electronics on the rabbit heart;^44^ (b) electrodes, a thermistor, and a heater on the balloon and in the catheter shaft;^107^ (c) deflation states of a polyurethane balloon with ablation and sensing electrodes;^13^ (d) an impedance contact sensor on a balloon;^43^ and (e) data from impedance sensor showing contact and no contact states.^43^

Kim *et al.* improved the ECG recording capability by implementing a flexible/stretchable, high-density, multiplexed array with active electrodes and temperature, pressure, and impedance sensors onto a balloon catheter.^13,43^ These high-density electrodes with a spatial density of 15 electrodes/cm^2^ are necessary, especially in the case of AF in which the electrical events of the heart are not well understood or defined, leading to reduced precision of the ablation treatment.^108,109^ With a greater number of electrodes, the need for more connections and wires could add bulk to the catheter shaft. Instead of taking that route, the group developed a distributed multiplexing circuit in which source followers (buffers) and complementary metal-oxide semiconductor (CMOS) switches that controlled the output of the active electrode. Using this strategy and controlling lines (rows/columns) of electrodes/amplifiers, a 64-electrode device was implemented on a balloon catheter with a reduced number (16) of inputs/outputs for high-density and high-resolution ECG recording. The impedance sensor [Fig. 4(d)] shows the expected performance, as shown in Fig. 4(e).

Implantable soft cardiac devices are necessary for real-time analysis, and *in vitro*/isolated cellular studies have provided insight into the function of the heart. However, functional behavior at the organ level has not been extensively studied, primarily due to the lack of competent tools, specifically for the *in vivo* case. Soft, multifunctional cardiac electronics can provide multimodal information that can assess fully remodeled disease states in order to augment the understanding of the heart’s functionality.^12^ Optical mapping, pH detection, temperature sensing, and strain sensing allow for a more comprehensive approach to invasive monitoring; these modes of sensing are discussed here.

Xu *et al.* printed heart models and fabricated three-dimensional-multifunctional integumentary membranes (3D-MIMs) that provided the capability to record electrophysiological signals, strain, optically map, and detect pH and temperature from the exterior surface of a perfused rabbit heart [Fig. 5(a)].^12^ Two-dimensional (2D) flexible sheets of semiconductor electronics were converted into 3D elastic membranes that mimic the shape of the heart. Fabrication involved 3D printing a rat heart model and development of the sensors on planar substrates. The sensors were transferred to a cured layer of silicone elastomer that surrounded the 3D-printed heart. The 3D-MIM consisted of μ-ILEDs (inorganic LEDs), strain gauges, sensing/stimulation electrodes, pH sensors, and temperature sensors/heaters. The membranes experienced pressures of the native pericardium.^110–115^ From their mechanical analysis, there was no indication of induced ischemia. They performed spatiotemporal cardiac measurements on a perfused rabbit heart. 68 Au electrodes (total surface area 1 mm^2^, 3.5-mm spacing) comprised part of the multifunctional sensor array. The array was placed over the epicardium and was transparent, allowing for optical mapping and verification of the ECG recording. The optical mapping data and electrical measurements were shown to be highly correlated [Fig. 5(b)]. The temperature sensor consisted of 16 serpentine traces made of Au. A linear response of the temperature sensor to the range of physiological temperatures was observed, and they had average responses of about 1.23 Ω/°C. The function of this sensor was tested by changing the temperature of the perfusion liquid and decreased temperatures (and consequently, decreased heart rate) were recorded with lower temperature perfusate [Fig. 5(c)]. This was confirmed with their ECG recording, which further indicated normal heart rhythms resuming after the original perfusion temperature was restored. 32 pH sensors were fabricated by depositing IrO_x_ on Au. Per unit of pH, a 68.9-mV potential response was recorded. pH mapping was demonstrated to track the progression of induced ischemia; they stopped the perfusion (passage of fluid) through the rabbit heart and observed that pH decreased from 7.4 (baseline) to 6.2, and then increased up to below the baseline value with reperfusion. Ventricular tachycardia was induced by this reperfusion, but eventually, the pH returned to baseline [Fig. 5(d)]. P-doped Si nanomembranes that exhibit piezoresistive behavior constituted the strain sensors. Three strain sensors arranged in a rosette layout had different purposes. Two sensors oriented in the ⟨110⟩ crystal direction characterized the rhythms of the heart, while one sensor oriented in the ⟨100⟩ direction was used for temperature calibration. In an experiment, they induced ventricular fibrillation with a drug (Pinacidil) and confirmed a random pattern from the strain gauges and abnormal waveforms in the ECG, which was expected for ventricular fibrillation [Fig. 5(e)]. The μ-ILEDs, based on AlInGaP, were used as light sources for optical mapping of fluorescent and voltage-sensitive dyes. Using the LEDs, cardiac action potentials were successfully recorded and a comparison with using an external light source verified this finding. Although passive electrodes were used in this study, active electrodes could provide clearer ECG signals.

**FIG. 5.**
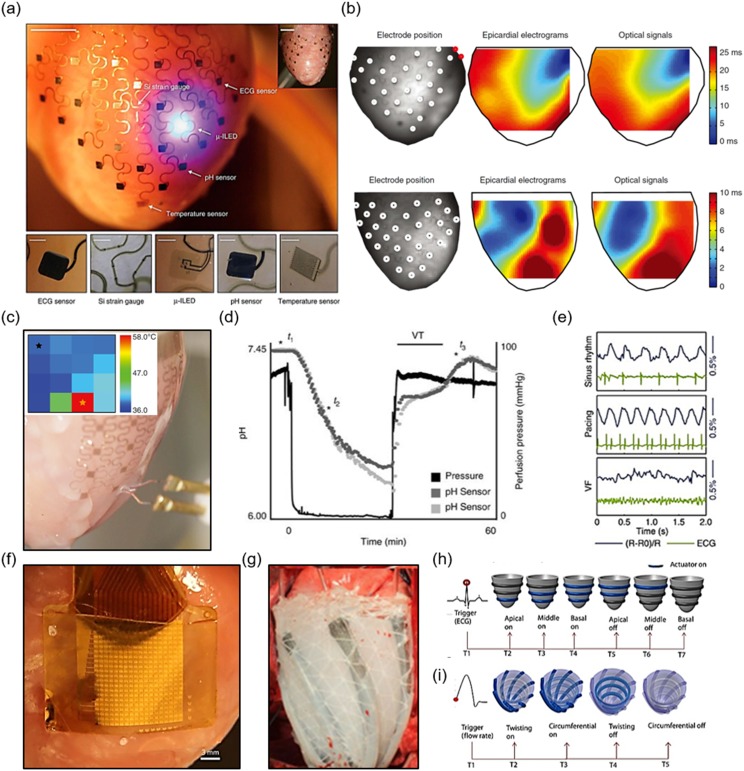
Multiparametric mapping with (a) ECG electrodes, μ-inorganic LEDs, pH sensors, temperature sensors, and strain sensors on the heart;^12^ (b) ECG and optical mapping comparison;^12^ (c) temperature mapping, inset shows the heat map during ablation;^12^ (d) pH detection during induced ischemia by stopping and resuming perfusion;^12^ (e) strain sensing with normal sinus rhythm, pacing, and ventricular fibrillation (VF);^12^ (f) array of capacitive sensors on *ex vivo* rabbit heart;^116^ (g) soft robotic sleeve with twisting actuators on porcine heart;^30^ (h) ECG control scheme for circumferential actuators, red dot indicates R peak trigger for actuation, gray actuators are not activated, and blue indicates actuation;^30^ and (i) aortic flow rate as the trigger for both radial and circumferential actuators, red dot indicates the start of increasing aortic flow.^30^

Fang *et al.* advanced cardiac electrophysiological mapping technology through the development of ultrathin capacitive, flexible, and multiplexed electronics that were durable in bio-fluids and conformally attached to the surface of the *in vitro* perfused rabbit heart [Fig. 5(f)].^116^ The advancements in this technology included leakage-free encapsulation that provided long-term stability in physiological conditions and high-resolution mapping of a dynamic surface using thin active electronics. The device had 396 multiplexed capacitive sensors that were 2500 *µ*m^2^ in size, where each sensor had two Si nanomembrane (Si NM) transistors, SiO_2_ that acted as both a dielectric and a barrier from the physiological environment, and PI as the encapsulation. The combination of the tissue interface with the SiO_2_ and Au contact pad of the transistor gate comprised the capacitor; it is important to note that this coupling of the semiconductor channel distinguished this device from conventional passive sensors.^117,118^ The sampling rate of this system was 1136 Hz per sensing node and could be improved by adjusting the multiplexing rate. Noise reached a maximum of ∼55 *µ*V and the signal-to-noise ratio (SNR) of the device was found to be 42 dB, which is excellent for heart monitoring.^119^ Mechanical analysis showed that the strain induced in the Si and SiO_2_ is much lower than their fracture limits. The encapsulation of this device was incredibly robust; the leakage levels were found to be 3 orders less than the safety limit standard for implanted medical devices. Polyvinyl chloride (PVC) improved the coupling of the device to the rabbit heart ventricles. The characteristic features of the ECG were discernable, and mapping of the voltage showed the phases of the cardiac action potential. The ECG was verified using optical methods, as the array was partially transparent. To demonstrate the functionality of the sensing array even in disease states, they showed that their phase maps could be used to identify ventricular fibrillation. The identification of the singularity in the phase map is a method used for ablation procedures.^120,121^ This technology offers a possible solution to map the Q-T interval (or repolarization of the heart) in QT syndrome studies that currently lack the appropriate tools. Wireless transmission of data from these types of sensors could allow for usage outside of hospitals.

Zhang *et al.* realized a flexible and stretchable magnetic strain sensor for wireless monitoring of cardiac strain on the pericardium of the heart.^122^ Laser-micromachined polymeric magnets were encapsulated by PDMS, which was further embedded into the softer Ecoflex. After fabrication, magnetization was performed using an induced magnetic field in a Halbach array, which strengthens the magnetic field on one side of the magnet while zeroing the field on the side. In their mechanical characterization, they found that although the bonding between the PDMS and magnetic stripe was not ideal, the PDMS still fixed the magnet in place. The Ecoflex served as a stretchable interconnect between the PDMS islands. The sensor was calibrated to successfully map physiologically relevant strains of the cardiac cycle, specifically 40%-60% with a range of 60-100 beats per minute (bpm). They mimicked cardiac cycle conditions and captured relevant signals using a smartphone application. Although this device can be used for passive monitoring, active devices, one of which is described here, could assist heart function.

Soft robotics is a burgeoning field in which mechanically soft materials are replacing the conventionally rigid materials used in the construction of robots. Walsh and co-workers created a soft robotic sleeve that can replace the conventionally bulky and rigid VADs used to temporarily sustain failing hearts [Fig. 5(g)].^30^ The tethered sleeve closely emulated the beating of the heart by replicating the helical and circumferential arrangement of cardiac muscle.^123–126^ These muscles both twist and compress during contraction. The fabricated sleeves employed a multilayered design that simulated the two outer muscle layers of the myocardium. The soft pneumatic artificial muscles (PAMs) were implemented in two designs, both of which conformally rested on the surface of the heart. Two designs were made. Design 1 was fabricated with two layers of silicon (Ecoflex) to make the individual actuators and the layers were combined; the final thickness was 16 mm. Design 2 was fabricated by selectively bonding two layers of silicone sheets in a predefined pattern and then combining them with layers of thermoplastic urethane (TPU) actuators, resulting in an overall thickness of 550 *µ*m. Computed tomography imaging confirmed the device’s conformability on the heart. A custom setup with ECG electrodes was developed to both record electrophysiological data along with confirming that the sleeve contracts in conjunction with the ex vivo heart. With various control schemes for the circumferential and twisting actuators, the sleeve synchronized with native heart motion; triggering of the actuation occurred with the ECG or hemodynamic parameters of the cardiac cycle [Figs. 5(h) and 5(i)]. Physiological volume displacement was a feature of this device that demonstrated relevance to the native cardiac environment. In a porcine model with acute heart failure, the robotic sleeve recovered the cardiac output from ∼45% of baseline up to 97% of baseline when activated. Further development of this device will require making it untethered, more miniaturized, and portable. In addition, this type of sleeve could deliver drugs to treat any patient’s heart condition and must also be further investigated to verify long-term stability and reduce the immune response.

Powering implantable devices has also come with various complications. Traditionally, surgery has been a requirement to replace any batteries used in implanted devices, bringing unnecessary risk and cost to the patient and caregiver.^127–130^ Although technology for batteries is constantly advancing, their relatively short operational lifetimes inhibit their usage as power supplies for devices that usually perform long-term operation, such as cardiac pacemakers. With a spontaneously functioning mechanical pump in the body, harvesting mechanical energy with implantable devices has transformed from an opportunity into a reality. Many strategies have been implemented to attempt to harness power from various processes in the human body.^131–138^ Yet, many of these devices fail to be mechanically compliant with the surface of the heart. The studies described below demonstrate functional energy harvesting and storage devices that conformally interface with the heart, through all its contractions and relaxations.

Dagdeviren *et al.* demonstrated the *in vivo* operation of a piezoelectric energy-harvesting device that was capable of concurrently generating and storing power from the contractions of the heart.^139^ The working mechanism of a piezoelectric material is as follows: external forces on the material induce electric fields within it.^140^ Where positive strain (stretch) occurs, a positive electrical potential can be detected, and where a negative strain (compression) occurs, a negative electrical potential can be detected. The main sensing element was a capacitor-like structure that had a layer of lead zirconate titanate (PZT) sandwiched between Ti/Pt and Cr/Au top and bottom electrodes, respectively [Fig. 6(a)]. The flexible mechanical energy harvester (MEH) consisted of 12 groups of this sensing element, each group containing 10 connected in parallel; this connectivity increased the output voltage. PI encapsulation isolated the device from leakage currents that can occur due to contact with bodily fluid and the device was shown to be stable in phosphate-buffered saline (PBS). The MEH was shown to be biocompatible in a cell viability test. When it experienced compression, the PZT and electrodes bent out of plane with the buckled substrate, and in an experimental simulation with different loads, higher frequency of bending led to more frequent current and voltage output. Even after 20 × 10^6^ bend and release cycles, the device did not show degradation in function. A chip-scale rechargeable battery and Schottky bridge rectifier stored the energy generated by the MEH. The device was sutured to 3 points of a bovine heart, specifically, the RV, the LV, and a free wall. From these points, the voltage output at the RV (∼4 V) was higher than that at the LV (∼3 V). This was attributed to the enhanced wall motion during contraction of the RV which allowed for greater bending of the MEH in comparison to the twisting motion of the LV during contraction. Exhale and inhale cycles were also recorded [Figs. 6(b) and 6(c)]. Numerous factors, such as the orientation of the MEH on the heart (angled placement), size of the heart, force of contraction, and heart rate were all considered when attempting to maximize output voltage. To ensure real-world applicability, the chest was closed, and the device was found to continue normal operation. Finally, by stacking multiple MEHs together and spin-casting silicone layers between them, they were able to generate a power density of up to 1.2 *µ*W/cm^2^, enough to power a cardiac pacemaker. In addition to taking advantage of the piezoelectric effect, the triboelectric effect has been shown to be effective for energy harvesting.

**FIG. 6.**
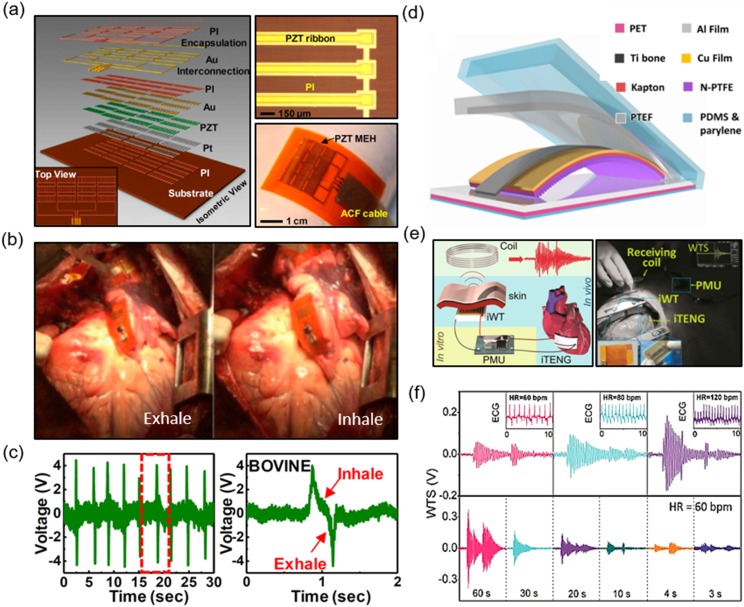
Piezoelectric energy harvester (a) schematic structure and device;^139^ (b) mechanical energy harvester (MEH) on a bovine heart during exhaling and inhaling;^139^ (c) data recorded showing exhale/inhale cycles;^139^ (d) schematic structure of a triboelectric nanogenerator (TENG);^141^ (e) custom wireless transmission system for cardiac monitoring;^141^ (f) wirelessly transmitted signals at different heart rates (top) and wirelessly transmitted signals at different charging times.^141^

Wang and co-workers implanted a triboelectric nanogenerator (TENG) for harvesting energy from the heartbeat of an *in vivo* porcine model and subsequently used it for cardiac monitoring.^141^ The working principle of TENG devices is as follows: by contacting two materials together, charge transfer occurs between them in a contact electrification process, leading to a deficit of electrons in one material (donor), while the other gains electrons (acceptor).^142^ Depending on the electrode setup used, alternating current will flow between the electrodes (to which each of the donor and acceptor is attached) or it will flow to ground (if a single electrode setup is used). This is also known as vertical contact separation mode. Their TENG device had a triboelectric layer of nanostructured polytetrafluoroethylene (n-PTFE), an ultrathin layer of Au deposited on a flexible substrate (Kapton film) which served as one electrode, and Al foil, which was the other electrode [Fig. 6(d)]. A strip of Ti was implemented to act as the adhesion support for the contact/separation of the TENG when the device was utilized *in vivo*. PDMS and parylene C encapsulated the entirety of the structure. Mouse fibroblasts were successfully cultured on the encapsulation material, proving that the device was biocompatible. *In vitro*, the TENG outputted an open circuit voltage of 45 V and a short circuit current of 7.5 *µ*A. After attaching a load resistance, the power density was found to be 107 mW/cm^2^. A capacitor was connected to the TENG, along with a rectifier, and it could be charged from 1.3 to 4.2 V in 150 s. In addition to measuring the output *in vivo* (with a porcine heart), the arterial pressure and ECG were recorded. The device was placed on the interior wall of the LV and an output of 14 V and 5 *µ*A was recorded. They were able to time synchronize the heart rate with this output; a capacitor was charged from 0.2 to 2.8 V with a heart rate of 80 bpm in 200 s. Like in the aforementioned paper, factors such as the location of placement, respiratory movement (diaphragm causing deformation of the pericardium), and cardiac contraction all impacted the output capability of the TENG. They created a self-powered wireless transmission system (SWTS) to monitor motion frequency of the heart [Fig. 6(e)]. An implantable wireless transmitter sent electromagnetic waves to an external receiving coil, which charged a capacitor. By analyzing the wirelessly transmitted power, they interpreted the frequency of beating and thus quantified the heart rate [Fig. 6(f)]. Again, the ECG verified the consistency of this observation. At 60 bpm, 3 s of charging was enough to send wireless data. Their long-term service evaluation showed that the device successfully operates for 3 days without hindering cardiac motion or causing any inflammatory reaction.

Soft cardiac bioelectronics have the potential to be fully integrated into the lifestyles of patients, as they will eventually allow the transmission of encrypted cardiac data from the patients’ homes to physicians. They will be used to identify the causes of pathophysiological conditions associated with the heart. Integrating these conformal devices with ubiquitous smartphones will boost their usability. This may lead to larger screening capabilities of communities and provide greater healthcare accessibility to those living in geographically isolated or economically limited areas.^143,144^ The omnipresence of machine learning ensures that these sensors could intelligently and continuously classify various cardiac events and immediately notify the user and caregiver of any critical conditions.

Soft bioelectronics for cardiac sensing and treatment is a growing area that needs future development. Some of the existing challenges involve the strict requirements of materials that (1) are resistive to corrosion, degradation, and electrical current leakage that may even induce disease states at the interfaced tissue and (2) are biocompatible to prevent immune responses when interfaced with the tissue.^145–147^ In addition, multifunctionality integrated in one device with the same mechanical properties is challenging yet critical for a more comprehensive understanding of the heart condition, since cardiac monitoring is not only limited to heart rate and ECG recordings.

Future directions for soft and wearable heart monitors will include multi-modal recording capabilities, integration of wireless and secure data transmission to enable remote and ambulatory monitoring, and incorporation of state-of-the-art machine learning technologies such as neural networks for providing enhanced feedback to users and their caregivers. The advances in engineering and science of materials and micromanufacturing techniques render the future scalability of these devices to make them more applicable to full human heart mapping and reducing the inflammatory response of the body to these devices. Replacing sensing elements with capacitive and soft sensors can circumvent the issues arising from metal electrodes. Integrated active electronics such as transistors may be utilized for higher density and greater resolution when recording electrophysiological data. Finally, power generators/supplies are indispensable components to be developed to power chronic implants.
